# Endoscopic diagnosis of gastric carcinoma of fundic gland type: A case report

**DOI:** 10.1097/MD.0000000000041575

**Published:** 2025-02-14

**Authors:** Chen Zhu, Wen Jiang, Zhaolian Bian

**Affiliations:** a Department of Gastroenterology and Hepatology, Nantong Third People’s Hospital, Affiliated Nantong Hospital 3 of Nantong University, Nantong, Jiangsu, China.

**Keywords:** endoscopic submucosal dissection, gastric carcinoma of fundic gland type

## Abstract

**Rationale:**

Gastric carcinoma of fundic gland type is a new type of gastric cancer with a low incidence rate. The mechanism of gastric carcinoma of the fundic gland differs significantly from that of common adenocarcinoma. While it typically presents as a benign growth, there are cases where it can progress to malignancy. If early endoscopy fails to provide an accurate diagnosis and the treatment is inappropriate, the tumor may metastasize and spread.

**Patient concerns:**

A 61-year-old male, abdominal discomfort, was performed gastroscopy.

**Diagnoses:**

Gastroscopy revealed a raised lesion approximately 1.8 cm × 1.5 cm in size in the middle of the gastric body, with surface flushing.

**Interventions:**

The patient was performed endoscopic submucosal dissection.

**Outcomes:**

The pathology showed gastric carcinoma of fundic gland type SM2, and the cure grade was eCura B. Follow-up review was conducted after communicating with the patient. After 6 months of follow-up, the patient shows no signs of recurrence or metastasis; future observations will require ongoing monitoring. This case emphasizes the difficulties of endoscopic diagnosis, and postoperative pathology confirmed the diagnosis of gastric carcinoma of the fundic gland type.

**Lessons:**

Gastric carcinoma of the fundic gland type is characterized by low atypia and easy SM invasion. This case highlights the importance of using endoscopy to identify this type, and select appropriate treatment methods. Because the infiltrative characteristics differ from those of conventional early gastric cancer, selecting endoscopic submucosal dissection treatment for lesions smaller than 2 cm can reduce postoperative tumor recurrence and metastasis.

## 1. Introduction

Gastric carcinoma of the fundic gland is a new type of gastric cancer with a low incidence rate. To date, no more than 100 cases have been reported in China. It is difficult to make a clinical endoscopic and pathological diagnosis. The authors reported a case of gastric fundus gland-type gastric cancer that was clearly diagnosed by combining endoscopy and pathology.

## 2. Case

**Figure 1.**
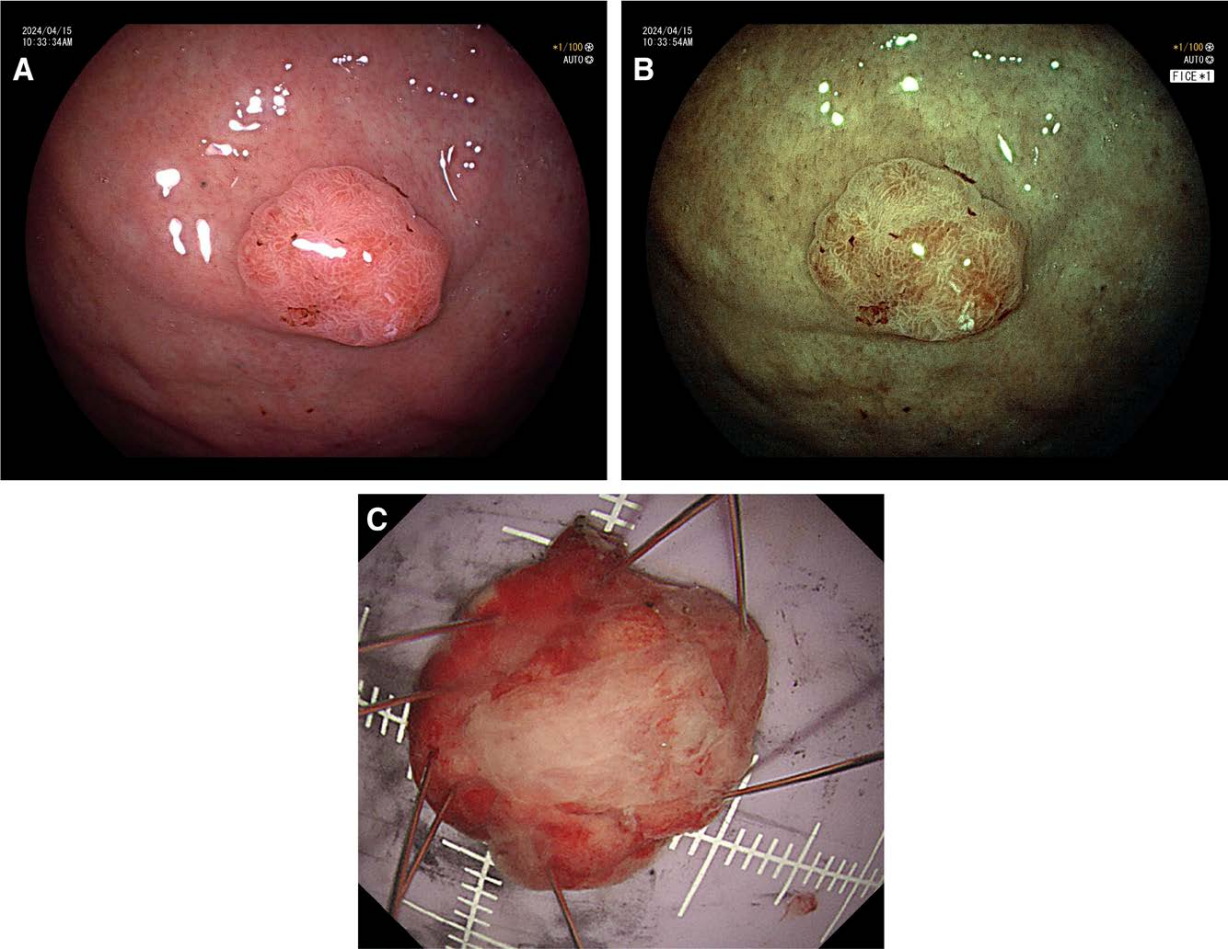
(A) A raised lesion about 1.8 cm × 1.5 cm in size was seen in the middle of the gastric body, with surface flushing. (B) FICE observed the lesion boundary was clear, and branched dilated blood vessels were seen on the surface. (C) ESD of gastric body. ESD = endoscopic submucosal dissection, FICE = flexible spectral imaging color enhancement.

**Figure 2.**
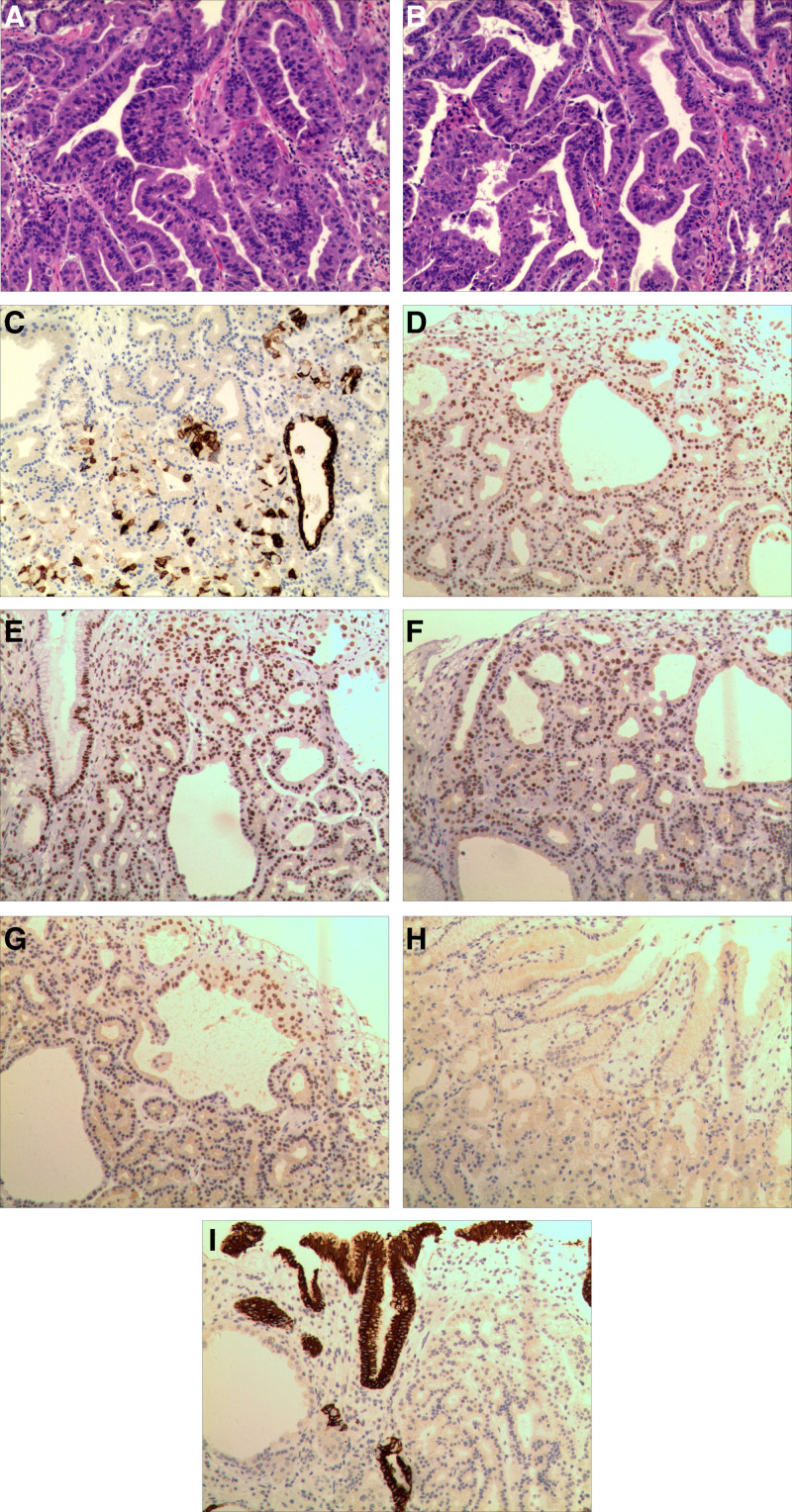
(A) Hyperplasia of oxyntic glands, mainly proliferation of main cells, scattered parietal cells. (B) Complex glandular structure, atypia of cells, and some areas break through the mucous muscle and infiltrate the submucosa. (C) Elastic fiber staining revealed vessels. (D) MSH2 (+). (E) MSH6 (+). (F) MLH1 (+). (G) PMS2 (+). (H) MUC2 (−). (I) MUC5AC (−).

A 61-year-old male, was admitted to the outpatient clinic for “abdominal discomfort for 1 month.” Gastroscopy showed multiple small polyps in the gastric body and fundus, and a raised lesion approximately 1.8 × 1.5 cm in size was seen in the middle of the gastric body, with surface flushing (Fig. 1A). Flexible spectral imaging color enhancement revealed that the lesion boundary was clear and branched dilated blood vessels were observed on the surface (Fig. 1B). Endoscopically, it was consistent with raised (IIa) and non-ulcerated (UL0) lesions. Endoscopic submucosal dissection (ESD) was performed. Postoperative pathological macroscopic findings: ESD of gastric body: a piece of mucosal tissue, 1.88 × 1.5 cm in size, a flat bulge mass on the mucosal surface, 1.78 × 1.4 cm in range, 0.3 cm higher than the mucosal surface, close to the incision margin (Fig. 1C); Microscopic findings: hyperplasia of oxyntic glands, mainly proliferation of main cells, scattered parietal cells (Fig. 2A), complex glandular structure, atypia of cells, and some areas break through the mucous muscle and infiltrate the submucosa (Fig. 2B). Immunohistochemistry (IHC-241336): wax tumor cells, no. MSH2 (+; Fig. 2D), MSH6 (+; Fig. 2E), MLH1 (+; Fig. 2F), PMS2 (+; Fig. 2G), MUC2 (−; Fig. 2H), MUC5AC (−; Fig. 2I), CDX-2 (−), Her-2 (−), CD31, and D2-40 showed vasculature, desmin showed mucosal muscle, P53 (wild type), Ki-67 (+, 3%), and Hp (−). Special staining: Elastic fiber staining revealed the vessels (Fig. 2C). Pathological diagnosis histological type: gastric fundus gland adenocarcinoma; tumor size: approximately 1.7 cm × 1.4 cm (see tumor tissue for articles 3–7); infiltration depth: infiltration of the submucosa, osmotic infiltration, cancer tissue infiltration of the anterior talar mucosa muscle 1100 µm (pT1b-SM2); vascular and nerve conditions: no vascular invasion (Ly0, V0), nerve invasion (−); presence or absence of ulcer: no ulcer formation (UL0); horizontal margin (lateral margin, pHM): no residual tumor pHM0 was found (the closest distance from the tumor was 1000 m); vertical margin (basal margin, pVM): no residual tumor pVM0 was found; peripheral gastric mucosa: chronic superficial inflammation; HP (−).

## 3. Discussion

Gastric adenocarcinoma of the fundic gland type was first reported by the Japanese scholar Tsukamoto^[1]^ in 2007, and the new edition in 2019 provided a definition of gastric tumor classification. Oxyntic gland adenoma was defined as a benign epithelial tumor accompanied by differentiation of the main cells and/or parietal cells, with the potential for submucosal invasion. Once submucosal invasion occurs, the oxyntic gland adenoma is called gastric carcinoma of the fundic gland, which is divided into 2 categories: low-grade dysplasia and high-grade dysplasia.

Gastric adenocarcinoma of the fundic gland type is characterized by low atypia and easy SM invasion. It mostly occurs in uninfected cases of HP, and most endoscopic manifestations are small lesions smaller than 1 cm. The lesions were mainly type 0-IIa. Most lesions faded, followed by congestive changes, and very few lesions were dark yellow or completely consistent with the background mucosa. Vascular dilation, which is considered a characteristic feature of endoscopy, is observed on the surface mucosa of most tumors.^[2–4]^

This case was of >1 cm IIa hyperemia. After ESD treatment, the pathology showed SM2, and the cure grade was eCura B. However, considering the low malignancy of fundus gland-type gastric cancer, a follow-up review was conducted after communication with the patient. The incidence of gastric fundus gland-type gastric cancer is not high and is mainly reported in East Asia. The risk factors and pathogenesis are unclear, and there are no clear guidelines to guide diagnosis and treatment. Recent studies suggest that the occurrence of gastric fundus gland polyps is related to the use of antacids.^[5]^ With the widespread use of antacids, the incidence of gastric fundus gland polyps is increasing and it is generally believed that gastric fundus gland polyps are less malignant. For lesions generally smaller than 2 cm, endoscopic treatment such as snare resection or endoscopic mucosal resection (EMR) is preferred. However, gastric fundic gland-type gastric cancer is prone to submucosal infiltration, and studies have shown that the 2-year recurrence rate after EMR in patients with gastric fundic gland-type gastric cancer is 27.27%,^[6]^ while the postoperative recurrence rate for ESD is 5.8%.^[7]^ It is necessary for endoscopists to early identify the lesion conditions through endoscopy, considering ESD treatment for gastric fundic gland polyps larger than 1 or 1.5 cm to reduce the risk of postoperative recurrence. Additionally, the endoscopic appearance of gastric fundic gland-type gastric cancer varies significantly, which may be related to different pathogenic mechanisms, and specific determinations still require more clinical case support.

## Author contributions

**Writing – original draft:** Chen Zhu, Wen Jiang.

**Writing – review & editing:** Zhaolian Bian.
